# Monochromatic Blue Light Activates Suprachiasmatic Nucleus Neuronal Activity and Promotes Arousal in Mice Under Sevoflurane Anesthesia

**DOI:** 10.3389/fncir.2020.00055

**Published:** 2020-08-18

**Authors:** Daiqiang Liu, Jiayan Li, Jiayi Wu, Jiaqi Dai, Xinfeng Chen, Yujie Huang, Shuang Zhang, Bo Tian, Wei Mei

**Affiliations:** ^1^Department of Anesthesiology, Tongji Hospital, Tongji Medical College, Huazhong University of Science and Technology, Wuhan, China; ^2^Wuhan National Laboratory for Optoelectronics, Huazhong University of Science and Technology, Wuhan, China; ^3^Chinese Institute for Brain Research (CIBR), ZGC Life Science Park, Beijing, China; ^4^Department of Neurobiology, Tongji Medical School, Huazhong University of Science and Technology, Wuhan, China; ^5^Key Laboratory of Neurological Diseases, Ministry of Education, Wuhan, China

**Keywords:** monochromatic blue light, polychromatic white light, suprachiasmatic nucleus, arousal, burst-suppression, electroencephalogram, sevoflurane

## Abstract

**Background**: Monochromatic blue light (MBL), with a wavelength between 400–490 nm, can regulate non-image-forming (NIF) functions of light in the central nervous system. The suprachiasmatic nucleus (SCN) in the brain is involved in the arousal-promoting response to blue light in mice. Animal and human studies showed that the responsiveness of the brain to visual stimuli is partly preserved under general anesthesia. Therefore, this study aimed to investigate whether MBL promotes arousal from sevoflurane anesthesia *via* activation of the SCN in mice.

**Methods**: The induction and emergence time of sevoflurane anesthesia under MBL (460 nm and 800 lux) exposure was measured. Cortical electroencephalograms (EEGs) were recorded and the burst-suppression ratio (BSR) was calculated under MBL during sevoflurane anesthesia. The EEGs and local field potential (LFP) recordings with or without locally electrolytic ablated bilateral SCN were used to further explore the role of SCN in the arousal-promoting effect of MBL under sevoflurane anesthesia. Immunofluorescent staining of c-Fos was conducted to reveal the possible downstream mechanism of SCN activation.

**Results**: Unlike the lack of effect on the induction time, MBL shortened the emergence time and the EEG recordings showed cortical arousal during the recovery period. MBL resulted in a significant decrease in BSR and a marked increase in EEG power at all frequency bands except for the spindle band during 2.5% sevoflurane anesthesia. MBL exposure under sevoflurane anesthesia enhances the neuronal activity of the SCN. These responses to MBL were abolished in SCN lesioned (SCNx) mice. MBL evoked a high level of c-Fos expression in the prefrontal cortex (PFC) and lateral hypothalamus (LH) compared to polychromatic white light (PWL) under sevoflurane anesthesia, while it exerted no effect on c-Fos expression in the ventrolateral preoptic area (VLPO) and locus coeruleus (LC) c-Fos expression.

**Conclusions**: MBL promotes behavioral and electroencephalographic arousal from sevoflurane anesthesia *via* the activation of the SCN and its associated downstream wake-related nuclei. The clinical implications of this study warrant further study.

## Introduction

General anesthesia can be described as a pharmacologically induced state of amnesia, immobility, unconsciousness, and analgesia (Taylor et al., 2016). It is expected that brain activities are silenced and it becomes unresponsive to external stimuli during deep general anesthesia (Ries and Puil, 1993; Detsch et al., 1999). However, animal and human studies have demonstrated that brain still responds to somatosensory (Yli-Hankala et al., 1993), auditory (Land et al., 2012), and visual stimuli (Hartikainen et al., 1995; Hudetz and Imas, 2007; Aggarwal et al., 2019) under general anesthesia. The underlying mechanisms of anesthetic actions remain unknown, while it has been suggested that anesthetic-induced loss of consciousness and sleep have shared neural mechanisms (Franks and Zecharia, 2011). Further comparison of natural sleep and general anesthesia may provide insights into the mechanisms of general anesthesia (Brown et al., 2010).

Light can modulate the sleep and wakefulness circuit and is involved in the image- and non-image-forming (NIF) visual functions in mammals (Legates et al., 2014). These NIF functions of light are mediated by melanopsin (OPN4), which is expressed in intrinsically photosensitive retinal ganglion cells (ipRGCs) that are projected to the suprachiasmatic nucleus (SCN) in the hypothalamus (Berson et al., 2002; Gooley et al., 2003). The NIF functions include entrainment of circadian rhythms to environmental light-dark cycles (Golombek and Rosenstein, 2010), regulation of pupil constriction (Gooley et al., 2012), alertness (Cajochen, 2007), cognition (Vandewalle et al., 2009), mood (Stephenson et al., 2012), and sleep and wakefulness (Altimus et al., 2008). Different wavelengths of light exert variable effects on sleep and arousal. Blue light enhances behavioral arousal *via* M1 ipRGC projection to the SCN in the hypothalamus in mice (Bourgin and Hubbard, 2016; Pilorz et al., 2016). In humans, exposure to monochromatic blue light (MBL) or blue-enriched polychromatic light in the evening has a more profound impact on non-rapid eye movement (NREM) and rapid eye movement (REM) sleep (Munch et al., 2006; Chellappa et al., 2013). As general anesthesia shares some features of the sleep-wake cycle (Vacas et al., 2013), a common finding between NREM sleep and anesthesia in imaging studies is the deactivation of the thalamus leading to cortical inhibition (Vacas et al., 2013). Modulation of certain nuclei or neurotransmitters that regulate the sleep-wake cycle can affect the systemic effects of general anesthesia (Taylor et al., 2016). Therefore, MBL may affect the induction, maintenance, or emergence of general anesthesia.

The SCN, acting as an information integration hub for light regulating the sleep-wake cycle, sends both monosynaptic and multisynaptic connections to hypothalamic nuclei implicated in the sleep-wake cycle (Mistlberger, 2005). Studies have confirmed that the SCN has a dense projection of the dorsomedial hypothalamus (DMH), which then sends inhibitory GABAergic projections to the ventrolateral preoptic area (VLPO) and excitatory glutamatergic projections to the lateral hypothalamus (LH; Chou et al., 2003). The DMH also projects to the locus coeruleus (LC), which sends excitatory output to wake-promoting neurons in the sleep/arousal network and cerebral cortex (Aston-Jones et al., 2001; Aston-Jones, 2005). Therefore, whether MBL affects general anesthesia *via* activation of the SCN and SCN downstream area remains unknown. To investigate the effect of MBL on sevoflurane general anesthesia and underlying mechanisms, we tested the time to lose of righting reflex (LORR) and recovery of the righting reflex (RORR) to explore the effect of MBL on anesthetic induction of and emergence from sevoflurane general anesthesia. Cortical electroencephalograms (EEG) were recorded, and the burst suppression ratio (BSR) and EEG power were calculated to assess the effect of MBL on sevoflurane general anesthesia. We employed immunofluorescent staining and local field potential (LFP) recordings to measure the changes in SCN activity induced by MBL under sevoflurane anesthesia. Bilateral ablation of the SCN was performed in mice to further confirm that the SCN plays a key role in the arousal-promoting effect of MBL under sevoflurane anesthesia. We observed the expression of c-Fos in the SCN’s typical projection areas during sevoflurane anesthesia under MBL exposure to explore the possible downstream mechanism of SCN activation.

## Materials and Methods

### Animals

All of the experiments were conducted following the National Institutes of Health’s Guidelines for the Care and Use of Laboratory Animals. All of the procedures were reviewed by the Experimental Animal Care and Use Committee of Tongji Medical College, Huazhong University of Science and Technology, China. Male C57BL/6J mice aged 10–12 weeks old and weighing 22–26 g were used throughout. They were housed in a facility with specific pathogen-free (SPF) conditions, a 12:12 light-dark cycle (lights on at 7:00 am and illumination intensity ≈ 300 lux), and food and water available *ad libitum*. All of the experiments were conducted during the daytime (8:00 am-5:00 pm).

### Design

**Figure 1:**
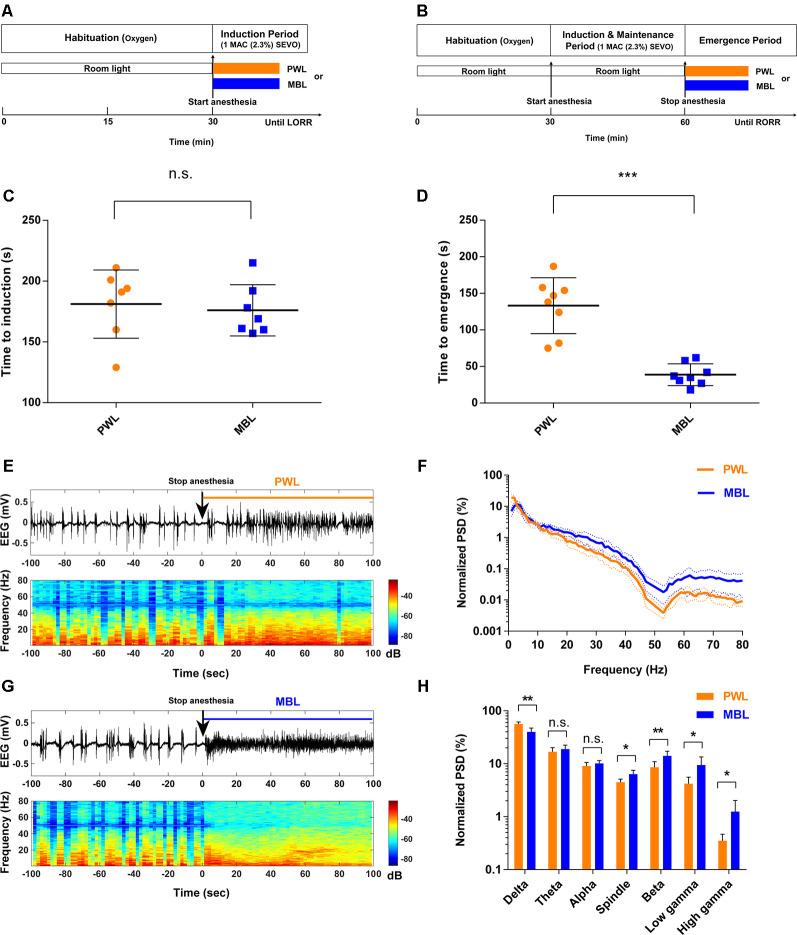
Monochromatic blue light (MBL) accelerated emergence from sevoflurane general anesthesia. **(A,B)** Timeline for light conditions administered during the induction **(A)** and recovery **(B)** periods with sevoflurane general anesthesia. **(C,D)** Induction **(C)** and emergence **(D)** times of anesthesia-induced by 1 minimum alveolar concentration (MAC; 2.3%) sevoflurane in the polychromatic white light (PWL) and MBL groups. MBL decreased the time to emergence (PWL, 133.10 ± 38.26 s, vs. MBL, 38.75 ± 14.96 s; ***p < 0.001 compared with PWL, *n* = 8 in each group, unpaired Student’s *t*-tests with Welch’s correction), while did not affect on the induction time (PWL, 181.10 ± 28.03 s vs. MBL, 176.00 ± 21.10 s) compared to PWL (n.s. = not significant compared with PWL, *n* = 7 in each group, unpaired Student’s *t*-tests with Welch’s correction). Data are expressed as mean ± SD. **(E,F)** Representative electroencephalogram traces (upper) and electroencephalogram power spectrogram (lower) in a mouse administered PWL **(E)** or MBL **(F)**. Time 0 indicates stopping sevoflurane anesthesia and the beginning of light stimulation. The orange and blue lines indicate the 100 s intervals used for power spectral density comparisons. **(G)** Normalized group power spectral densities (PSD) in the PWL (orange) and MBL (blue) groups. **(H)** MBL decreased the relative electroencephalogram delta power and increased the spindle, beta, low gamma, and high gamma power compared to PWL (*p < 0.05, **p < 0.01 compared with PWL, n.s. = not significant, *n* = 6 in each group, unpaired Student’s *t*-tests with Welch’s correction). Data are expressed as mean ± SD. PWL, polychromatic white light; MBL, monochromatic blue light.

**Figure 2:**
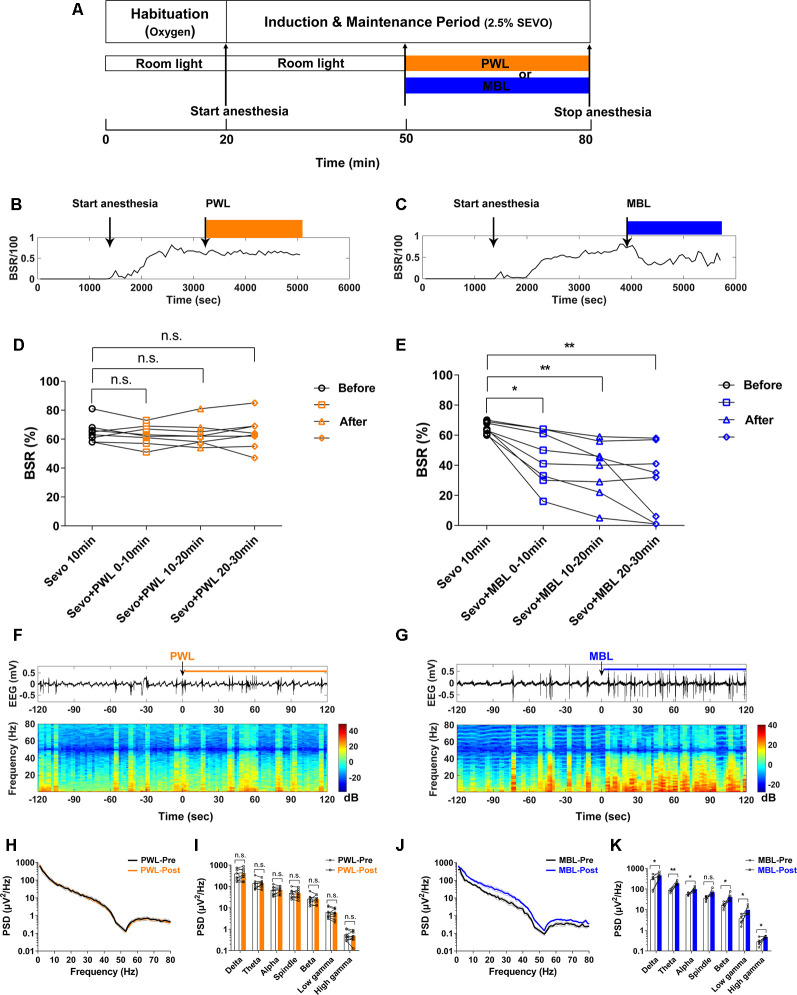
MBL decreased the burst suppression ratio (BSR) during continuous inhalation of 2.5% sevoflurane. **(A)** Timeline for light conditions administered in the burst suppression period during continuous inhalation of 2.5% sevoflurane. **(B,C)** Typical examples of the BSR response under sevoflurane anesthesia in a mouse administered PWL **(B)** or MBL **(C)**. The orange and blue boxes indicate the duration of light exposure. **(D,E)** Effect of PWL **(D)** and MBL **(E)** on electroencephalogram BSR during continuous inhalation of 2.5% sevoflurane. Mice treated with MBL showed a significant 31% decrease (*) in the BSR during the first 10 min post-MBL, a 42% decrease (**) during the second 10 min post-MBL, and a 55% decrease (**) during the last 10 min post-MBL compared to the pre-MBL 10 min (*p < 0.05, **p < 0.01 compared with pre-MBL 10 min, *n* = 8, RM one-way ANOVA, and Tukey’s multiple comparisons test with the Greenhouse-Geisser correction, *F*_(2.007,14.05)_ = 16.18). However, PWL-treated animals showed no significant difference in the BSR compared to pre-PWL (n.s. = not significant, compared with pre-PWL 10 min, *n* = 8, RM one-way ANOVA, and Tukey’s multiple comparisons test with the Greenhouse-Geisser correction, *F*_(2.540,17.78)_ = 0.5143). **(F,G)** Representative electroencephalogram traces (upper) and electroencephalogram power spectrogram (lower) in a mouse administered PWL **(F)** or MBL **(G)**. Time 0 indicates the beginning of light stimulation during continuous sevoflurane anesthesia. The orange and blue lines indicate the 120 s intervals used for power spectral density comparisons. **(H)** Grouped PSD computed from 2 min before PWL (black) and 2 min after PWL (orange). **(I)** PWL-treated animals showed no statistically significant difference in electroencephalogram power compared to pre-PWL (n.s. = not significant, compared with pre-PWL, *n* = 9, and paired Student’s *t*-tests). **(J)** Grouped PSD computed from 2 min before MBL (black) and 2 min after MBL (blue). **(K)** MBL-treated animals increased electroencephalogram power in all of the frequency bands except for the spindle band compared to pre-MBL (*p < 0.05, **p < 0.01 compared with pre-MBL, n.s. = not significant, *n* = 6, and paired Student’s *t*-tests). PWL, polychromatic white light; MBL, monochromatic blue light.

Two light wavelength conditions, MBL (460 nm, MBL; LS, Jiadeng, China) and polychromatic white light (PWL, Supplementary Figure S1; 12-LE-41490, OPPLE, China) were used in our study. Figures 1A,B, 2A illustrate the experimental design. For the loss of righting reflex (LORR) study, MBL (460 nm, 800 lux) or PWL (800 lux) was administered when sevoflurane anesthesia was initiated, and then the light exposure will continue until LORR occurred. For the return of the righting reflex (RORR) study, MBL (460 nm and 800 lux) or PWL (800 lux) was administered when sevoflurane anesthesia ceased, and then the light exposure will continue until RORR occurred. For the EEG or LFP recording study during the burst suppression period, MBL (460 nm and 800 lux) or PWL (800 lux) was administered along with anesthesia for 30 min after the mice were under steady-state sevoflurane anesthesia. For the EEG recording study during the emergence period, MBL (460 nm and 800 lux) or PWL (800 lux) was administered when sevoflurane anesthesia stopped, and then the light exposure will continue until RORR occurred.

### Loss and Return of Righting Reflex

Induction of and emergence from sevoflurane are defined behaviorally as the respective loss and return of the righting reflex (Kelz et al., 2008). Thus, to assess sevoflurane induction and emergence, the time to loss of the righting reflex (LORR) and time to recovery of the righting reflex (RORR) were investigated. The mice were placed in an open-circuit rodent anesthesia system containing eight isolated transparent cylindrical anesthesia chambers (12.5 cm in length and 5 cm in diameter). After 2 h of habituation with 0.5 L/min 100% oxygen each day on three successive days, anesthesia was induced with a sevoflurane vaporizer (RWD, Shenzhen, China) in concentrated anesthetic gas dissolved in 100% oxygen. The concentration of sevoflurane in this system was monitored continuously with an anesthetic agent analyzer (G60, Philips, Shenzhen, China). To measure the time to LORR, each mouse was placed individually in the small chamber, after which 1 minimum alveolar concentration (MAC; 2.3%) of sevoflurane was delivered with 0.5 L/min oxygen. At 15-s intervals, the chamber was rotated to place the mouse on its back, and the ability to right itself was assessed. A mouse was considered to have lost the righting reflex if it did not turn itself prone onto all four limbs more than 30 s. The time to LORR (induction time) was defined as the time at which a mouse first lost its righting reflex from the time of sevoflurane onset. After 30 min of exposure to 1 MAC (2.3%) of sevoflurane, the mouse was immediately removed from the chamber and placed in a supine position in room air. A mouse was considered to have recovered the righting reflex if it turned itself prone onto all four feet on the floor. The time to RORR (emergence time) was defined as the time at which a mouse regained its righting reflex from the time of removal from the sevoflurane chamber. The mouse temperature was maintained between 36.6 ± 0.6°C by putting the cylindrical chambers on a far-infrared warming pad (RightTemp, Kent Scientific, Torrington, CT, USA) that had been warmed to 37°C.

### Surgical Placement of Electroencephalogram Electrodes and Intracranial Electrodes

The mice were anesthetized with 1% sodium pentobarbital (50 mg/kg, i.p.). The animals were placed in a stereotaxic apparatus (RWD, Shenzhen, China). A heating pad (RWD, Shenzhen, China) was used to maintain stable body temperature. Erythromycin eye ointment was applied to their eyes. With additional subcutaneous bupivacaine for analgesia, a longitudinal incision was made along the midline skull, and blunt dissection was performed to expose the suture lines of the skull. The EEG electrode consisted of a 0.2 mm diameter silver wire and an M 0.6 * 1.5 stainless steel screw. Two stainless steel screws were fixed in the right frontal cortex (0.4 mm lateral and 1.75 mm anterior to the Bregma) and left cerebellum for EEG recording (Liu et al., 2020). All of the leads were connected to a miniature plug. Intracranial LFP electrodes (PFA-coated stainless steel wire, 139.7 μm coated diameter; Cat. No. 791000; A-M Systems, Sequim, WA, USA) were positioned in the left SCN (0.46 mm posterior to the Bregma, 0.1 mm lateral to the midline, and 5.3 mm ventral to the surface of the cortex). Silver wires (99.99%, 0.2 mm diameter, China) were anchored with screws in the right cerebellum as reference electrodes. The EEG and LFP electrodes were secured to the skull with Super-Bond C&B and dental acrylic. After surgery, the mice were kept on a heated sheet for complete recovery from anesthesia. All of the animals that underwent surgery were kept on a standard light-dark cycle for at least 7 days before anesthesia and EEG and LFP recording.

### EEG Analysis

The EEG signal was amplified at a gain of 1,000, analog bandpass-filtered at 0.1–500 Hz using a Model 1700 differential alternating-current amplifier (A-M Systems, Sequim, WA, USA), and collected with a sampling frequency of 500 Hz using a PCIe 6323 data acquisition board (National Instruments, Austin, TX, USA). The EEG signals were continuously recorded with Spikehound software (Lott et al., 2009). Large artifacts in the EEG were only seen during the gross motor movement. We ascertained recorded periods free from such events and artifact-free EEG data were selected for analysis (EEG recording during BSR period, the data in three mice was omitted in group MBL, the data in four mice was omitted in group Sham + PWL).

#### EEG Burst Suppression Identification and BSR Calculation

The raw EEG data were preliminarily band-pass filtered (10–80 Hz) and band-block filtered (48–52 Hz) to remove line noise. Each EEG recording was detrended and smoothed by convolution with a Gaussian function. The burst suppression ratio (BSR) was calculated by segmenting the EEG into bursts and suppressions using a voltage- and duration-based threshold (Chemali et al., 2011). Suppression is commonly defined as an interval in which the amplitude of the time-differentiated EEG signal remains within a −15 to 15 μV/s window for at least 200 ms, and suppression events are considered parts of the same suppression when the inter-event interval is ≤50 ms. EEG epochs between suppression periods were considered as bursts. For the BSR algorithm, suppressions were given a value of 1, and bursts were given a value of 0 to create a binary time series. This binary time series was then smoothed with a windowing function to calculate the BSR over time (Vijn and Sneyd, 1998). The raw EEG signals were exported into MATLAB R2019a (MathWorks, Natick, MA, USA) and analyzed using appropriate codes.

#### EEG Power Spectral Analysis

We computed spectrograms using multitaper methods from the Chronux toolbox (version 2.1.2)^1^ in MATLAB R2019a (MathWorks, Natick, MA, USA). The spectrogram was displayed using a decibel scale. Areas with warm colors indicated high spectral power while cool colors indicated low spectral power; 120 s (for the burst-suppression period) or 100 s (for the emergence period) of spontaneous brain activity was computed every 4 s (50% overlapping), band-pass filtered (1–80 Hz), and band-block filtered (48–52) to remove line noise, followed by 5-tapers fast Fourier transform (FFT). For the emergence period, grouped power spectral densities (PSD) were normalized by the average power at each frequency point from 1 to 80 Hz to obtain a unitless ratio. For the burst-suppression period, the “before” and “after” groups’ PSD were mean PSD over the entire frequency band (1–80 Hz) and for single-frequency bands of raw EEG signals. The averaged power values were calculated for the following frequency bands: delta (1–4 Hz), theta (4–8 Hz), alpha (8–12 Hz), spindle (12–15 Hz), beta (15–25 Hz), low gamma (25–48 Hz), and high gamma (52–80 Hz).

### LFP Analysis

Only signals from electrodes localized to the SCN were included to analyze the LFP. LFP signals from the SCN were amplified at a gain of 1,000, analog bandpass-filtered at 0.1–500 Hz using a Model 1700 differential alternating-current amplifier (A-M Systems, Sequim, WA, USA), filtered (1–80 Hz), digitized at 1,000 Hz, and stored for offline analysis. Only high-quality data devoid of artifacts were selected for analysis (data in 1 mouse was omitted for LFP recording in group MBL). LFP changes within 120 s before and after light administration were analyzed. LFP-averaged PSD over the entire frequency band (1–80 Hz) was computed *via* Welch’s method using 4 s segments and a Hanning window with a 50% overlap, followed by 5-taper FFT. To minimize the impact of interference from external electrical sources, power at 48–52 Hz was excluded from the analysis.

### Histological Analysis

After LFP recording, all of the animals were sacrificed for histological analysis. The mice were deeply anesthetized with 1% sodium pentobarbital (100 mg/kg, i.p.), then perfused with phosphate-buffered saline (PBS; 1X) followed by 4% ice-cold paraformaldehyde (PFA). The brains were fixed by 4% PFA at 4°C for at least 48 h, then removed and post-fixed in 4% PFA at 4°C for 24 h and dehydrated in 30% sucrose in PBS at 4°C until they sank. The brain tissue was serially sectioned at 40 μm on a freezing microtome (CM1900, Leica, Germany). The sections were mounted on glass slides and stained with DAPI. Images were captured using a fluorescence microscope (DM2500, Leica, Germany) and overlaid with best-fitting sections from the Mouse Brain in Stereotaxic Coordinates (Paxinos and Franklin, 2001). The end of the track (tissue scarring where the electrode was placed) was used to determine the electrode location.

### SCN Lesions

Bilateral ablation of the SCN was performed before the electroencephalogram electrode implantation according to previously described methods (Coomans et al., 2013). The mice were anesthetized and skin incisions were performed according to the previously described protocol. A hole was drilled through which the lesion electrode was inserted into the brain. A sheathed platinum-iridium electrode with a 0.2 mm exposed tip (KD-SCS-Pt/Ir-DM40-LE50, Kedoubc, Suzhou, China) was aimed at the SCN 0.46 mm posterior to the Bregma, 0.1 mm lateral to the midline, and 5.3 mm ventral to the surface of the cortex (Paxinos and Franklin, 2001). The lesion electrode was connected to the stimulator’s positive pole, while the stimulator’s negative pole extension cord was fixed on the mouse scalp. Bilateral electrolytic SCN lesions were produced using an isolated pulse stimulator (model 2100, A-M Systems, Sequim, WA, USA) to generate a 2.5 mA current pulse for 20 s. The sham lesion mice underwent the same operation, but no current was passed through the electrode. After recovering from surgery, the mice were given a vision test to detect potential surgical damage to the optic nerve. Each animal was placed in a large glass-fronted box with obstacles. Movements and reactions to the obstacles were observed and recorded for 10 min by two independent observers blinded to the allocated groups. Only the mice that showed normal vision were included in the SCN lesioned group. To verify the position of the SCN lesions, the surviving lesioned mice were deeply anesthetized after the study with 1% sodium pentobarbital (100 mg/kg, i.p.) and perfused with PBS (1X) followed by 4% ice-cold PFA. Brains were removed and fixed by 4% PFA at 4°C overnight, then dehydrated in 30% sucrose in PBS at 4°C until they sank. The brain tissue was serially sectioned at 40 μm on a freezing microtome (CM190, Leica, Germany). Sections were mounted on glass slides and stained with Nissl stain.

### Immunofluorescent Staining and Cell Counting

The mice were placed in acrylic glass chambers with inflow of 100% oxygen. After 2 h of conditioning, 2.5% sevoflurane was administered to the chamber for 30 min, and the same luminance PWL and MBL (460 nm) were administered to the mice for 30 min, respectively. After the light illumination was complete, the mice were immediately deeply anesthetized with 1% sodium pentobarbital (100 mg/kg, i.p.) and perfused intracardially with PBS (1×) followed by 4% ice-cold PFA. After perfusion, the brains were harvested, post-fixed in 4% PFA at 4°C for 4 h, and dehydrated in 30% sucrose in PBS at 4°C until they sank. The brains were coronally sectioned into 20 μm slices on a freezing microtome (CM1900, Leica, Germany), mounted on poly-lysine-coated slides, and stored at −80°C until use.

For c-Fos staining, the brain sections were penetrated with 0.3% Triton-X-100 for 15 min and blocked with 10% goat serum for 1 h at room temperature and incubated overnight at 4°C with rabbit anti-c-Fos antibody (1:500, Cat. No. 226 003, Synaptic Systems, Germany). The sections were then incubated with DyLight 594 and goat anti-rabbit secondary antibody (1:300, A23420, Abbkine, CA, USA) for 2 h at room temperature and washed with PBS. The sections were then covered with DAPI (AR1177, Boster, Wuhan, China) for 5 min at room temperature and rinsed, mounted, and cover-slipped with 50% glycerol. For double immunofluorescence, the sections were incubated with a mixture of rabbit anti-c-Fos antibody (1:500, Cat. No. 226 003, Synaptic Systems, Germany) and human/mouse tyrosine hydroxylase antibody (1:100, Cat. No. MAB7566, R&D Systems, USA) or human/mouse/rat orexin-A/hypocretin-1 antibody (1:100, Cat. No. MAB763, R&D Systems, USA) overnight at 4°C. The sections were then incubated with a mixture of DyLight 594, goat anti-rabbit secondary antibody (1:300, A23420, Abbkine, CA, USA), DyLight 488, and goat anti-mouse secondary antibody (1:200, A23210, Abbkine, CA, USA) for 2 h at room temperature and washed with PBS. The sections were rinsed, mounted, and cover-slipped with 50% glycerol. Images were captured using a fluorescence microscope (DM2500, Leica, Germany). The c-Fos positive cells were counted in alternate sections in the SCN nucleus approximately −0.22 to −0.82 mm from the Bregma. VLPO c-Fos positive cell counts were performed on sections spanning from +0.26 to −0.10 mm relative to the Bregma using a standardized 400 × 250 μm box positioned 300 μm lateral to the midline (Moore et al., 2012; Han et al., 2014). The TH c-Fos-positive cells were counted on alternate sections in the locus coeruleus (LC) approximately −5.20 to −5.80 mm from the Bregma. The numbers of orexin c-Fos-positive neurons were counted in alternate sections in the lateral hypothalamus (LH; approximately −0.34 to −2.80 mm from the Bregma) and prefrontal cortex (PFC; approximately from 2.34 to 1.54 mm from the Bregma).

### Statistical Analysis

The results were presented as mean ± standard deviation (SD). Statistical analyses were conducted using GraphPad Prism 6 (GraphPad Software, San Diego, CA, USA). All of the data for statistical analysis were first assessed for a normal distribution using the Kolmogorov–Smirnov test. Non-normally distributed data were log-transformed before statistical analysis to ensure normality and homogeneity of variance. Group comparisons in repeated measures design (BSR) were tested with repeated measures (RM) one-way ANOVA and Tukey’s multiple comparisons test was applied for multiple hypothesis testing. The Greenhouse-Geisser correction was applied when sphericity could not be assumed. Group differences in c-Fos numbers, behavior results (time to induction and time to emergence), and normalized EEG PSD during the emergence period were compared using independent samples unpaired Student’s *t*-tests with Welch’s correction. After the SCN lesioned, group comparisons of behavior results (time to induction and time to emergence) and normalized EEG PSD during the emergence period were analyzed using ordinary one-way ANOVA followed by Tukey’s multiple comparisons test. Differences in LFP PSD and EEG PSD during the burst suppression period between the pre- and post-events were analyzed using paired Student’s *t-tests*. In all of the cases, a *p* < 0.05 was considered statistically significant.

## Results

### Monochromatic Blue Light Accelerated Emergence From Sevoflurane General Anesthesia

Figures 1A,B illustrate the experimental protocol used to test the effect of MBL on the time to induction and time to emergence from sevoflurane anesthesia. For the LORR study, before sevoflurane anesthesia, the mice were exposed to room light (regular fluorescent bulbs) in cylindrical glass anesthesia chambers. When sevoflurane exposure started, the same illumination intensity (800 lux) of MBL or PWL was administered on the acrylic glass chamber surface devoid of any other source of light, respectively (Supplementary Figure S2). The time to LORR under MBL or PWL exposure was then recorded (Figure 1A). For the RORR study, the mice were under anesthesia for 30 min and exposed to room light. When sevoflurane exposure stopped, the same illumination intensity (800 lux) of MBL or PWL was administered and then the time to RORR under MBL or PWL was recorded (Figure 1B). As shown in Figure 1C, the mice that were exposed to MBL (176.00 ± 21.10 s) showed no statistically significant difference in the time to induction compared to the PWL group (181.10 ± 28.03 s, *p* = 0.7055, *t* = 0.3878, and *df* = 11.15). However, a significant difference in recovery time was found between the PWL and MBL groups (133.10 ± 38.26 s vs. 38.75 ± 14.96 s, *p* < 0.001, *t* = 6.497, and *df* = 9.092; Figure 1D), indicating that MBL could lead to a faster emergence from sevoflurane anesthesia.

To further evaluate how MBL could affect arousal, we analyzed cortical EEG activity for a duration of 100 s after stopping sevoflurane anesthesia. We observed a faster transition from an anesthesia-EEG state to a wake-EEG state after MBL treatment compared to the PWL group during the sevoflurane emergence period (Figures 1E,F). Spectral analysis of the EEG data for a duration of 100 s after sevoflurane stopped showed that MBL decreased relative to the electroencephalogram delta power (*p* = 0.0014, *t* = 4.535, and *df* = 9.058) and increased relative to the spindle (*p* = 0.0102, *t* = 3.383, and *df* = 7.657), beta (*p* = 0.0077, *t* = 3.398, and *df* = 9.141), low gamma (*p* = 0.0213, *t* = 3.067, and *df* = 6.157), and high gamma (*p* = 0.0361, *t* = 2.804, and *df* = 5.217) power, while the theta (*p* = 0.3399, *t* = 1.002, and *df* = 9.934) and alpha (*p* = 0.2249, *t* = 1.294, and *df* = 9.945) band power remained unchanged (Figures 1G,H), indicating that MBL accelerated cortical arousal during the sevoflurane emergence period. These findings demonstrate that MBL could promote behavioral arousal from sevoflurane anesthesia and accelerate cortical arousal during the sevoflurane emergence period.

### Monochromatic Blue Light Decreased the Burst Suppression Ratio During Continuous Inhalation of 2.5% Sevoflurane

Figure 2A illustrates the experimental protocol used to investigate the effect of MBL on the burst suppression ratio (BSR) during continuous inhalation of 2.5% sevoflurane. After the mice were under anesthesia for 30 min in room light in cylindrical glass anesthesia chambers, they were continually anesthetized and administered at the same illumination intensity (800 lux) of MBL or PWL for 30 min. As shown in Figures 2B,C, typical examples of the BSR response during continuous inhalation of 2.5% sevoflurane showed that MBL decreased the BSR, while PWL did not. The mice treated with MBL showed a significant 31% decrease (*p* < 0.05) in the BSR during the first 10 min post-MBL and a 42% decrease (*p* < 0.01) during the second 10 min post-MBL and a 55% decrease (*p* < 0.01) during the last 10 min post-MBL compared to the pre-MBL 10 min (Figure 2E, *F*_(2.007,14.05)_ = 16.18). However, the PWL-treated animals showed no significant difference in their BSR compared to pre-PWL (Figure 2D, *F*_(2.540,17.78)_ = 0.5143, *p* > 0.05).

A representative EEG recorded during continuous inhalation of 2.5% sevoflurane (Figure 2G) showed that MBL changed the burst suppression pattern to more burst occurrence. A spectrogram computed from the same animal in Figure 2G showed that the EEG power after MBL administration was higher than pre-MBL. However, there was no change in the electroencephalogram amplitude or frequency and power between pre-PWL and post-PWL (Figure 2F). The combined PSD computed from 120 s before and after MBL administration in the six animals (Figures 2J,K) showed that the power at all of the frequency bands except for the spindle band significantly increased after MBL administration (*p* < 0.05), indicating that MBL could increase EEG activation during continuous deep anesthesia. The PWL-treated animals showed no statistically significant difference in electroencephalogram power compared to pre-PWL (Figures 2H,I, *p* > 0.05). These findings demonstrated that MBL could decrease the depth of anesthesia and increase cortical EEG activity during continuous deep sevoflurane anesthesia.

### Monochromatic Blue Light Drove Suprachiasmatic Nucleus Activation During Sevoflurane Anesthesia

**Figure 3:**
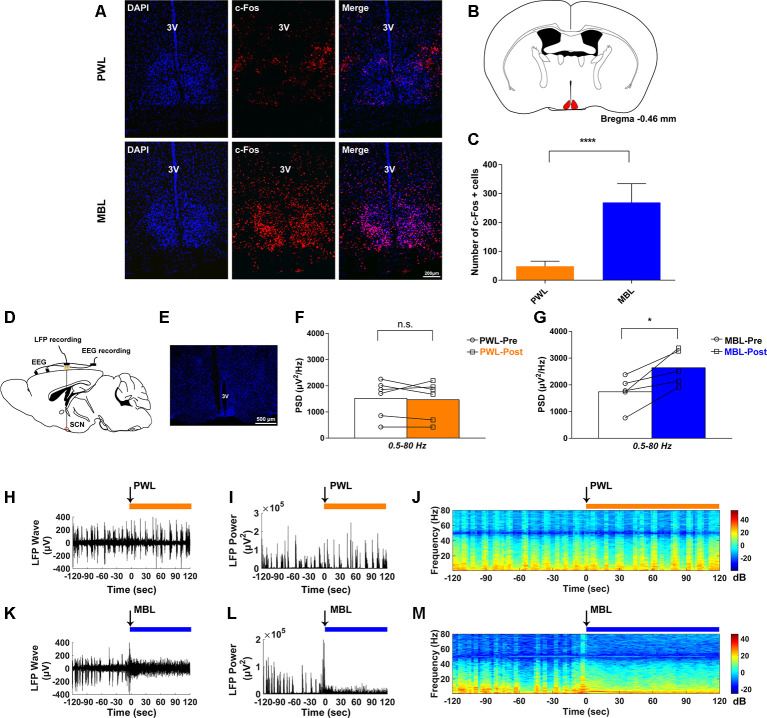
MBL drove suprachiasmatic nucleus (SCN) activation during sevoflurane anesthesia. **(A)** Effect of PWL (upper) and MBL (lower) on c-Fos immunostaining and DAPI nuclear staining in the SCN during sevoflurane anesthesia. 3V, third ventricle. Scale bar = 200 μm. **(B)** Position of the SCN in the mouse brain atlas. The red indicates the SCN. **(C)** Quantitative analysis of the number of c-Fos-positive cells in the PWL and MBL groups. c-Fos expression in the SCN region with MBL treatment was significantly higher than in the PWL treatment group (****p < 0.0001 compared with PWL, *n* = 8 in each group, and unpaired Student’s *t*-tests with Welch’s correction). Data are expressed as mean ± SD. **(D)** Schematic of the experiment. EEG and local field potential (LFP) in the SCN were simultaneously recorded. **(E)** The coronal section containing the track of an electrode localized to the SCN. 3V, third ventricle. Scale bar = 500 μm. **(F)** Quantitative spectral analysis computed from 2 min before PWL (white) and 2 min after PWL (orange) on the entire frequency band (1–80 Hz). The PWL-treated animals showed no statistically significant difference in the SCN LFP power compared to pre-PWL (n.s. = not significant compared with pre-PWL, *n* = 6, and paired Student’s *t*-tests). **(G)** Quantitative spectral analysis computed from 2 min before MBL (white) and 2 min after MBL (blue) on the entire frequency band (1–80 Hz). The MBL-treated animals increased the SCN LFP power compared to pre-MBL (*p < 0.05 compared with pre-MBL, *n* = 5, and paired Student’s *t*-tests). **(H–J)** Representative SCN LFP traces **(H)**, LFP power **(I)**, and LFP power spectrogram **(J)** in a mouse administered PWL. Time 0 indicates the beginning of light stimulation during continuous sevoflurane anesthesia. The orange lines indicate the 120 s intervals used for power spectral density comparisons. **(K–M)** Representative SCN LFP traces **(K)**, LFP power **(L)**, and LFP power spectrogram **(M)** in a mouse administered MBL. Time 0 indicates the beginning of light stimulation during continuous sevoflurane anesthesia. The blue lines indicate the 120 s intervals used for power spectral density comparisons. PWL, polychromatic white light; MBL, monochromatic blue light.

To determine whether the SCN was involved in effects induced by MBL exposure under sevoflurane anesthesia, we examined immunofluorescent expression of c-Fos, a marker of antecedent neuronal activity. Figures 3A,C show that MBL exposure under sevoflurane anesthesia elicited a significant increase in c-Fos expression in the SCN (Figure 3B, the red indicates SCN) compared to PWL treatment groups (*p* < 0.0001, *t* = 9.276, and *df* = 8.027), suggesting that MBL exposure under sevoflurane anesthesia could enhance SCN neuron activity.

To further evaluate whether the neural activity was affected by MBL, we simultaneously recorded both LFP at the SCN and cortical EEG activity through EEG recordings (Figure 3D). We used DAPI staining for histological analysis. The track in Figure 3E was used to determine the electrode location. Consistent with the immunofluorescent staining results, SCN LFP activity increased after the application of MBL compared to pre-MBL. This was demonstrated by the total power of entire frequency band (1–80 Hz) in the SCN, which significantly increased after the application of MBL (*p* = 0.0189, *t* = 3.813, and *df* = 4), while there was no statistically significant difference in the SCN LFP total power of the entire frequency band (1–80 Hz) between pre-PWL and post-PWL (*p* = 0.7144, *t* = 0.3874, and *df* = 5; Figures 3F,G). As shown in Figures 3H–J, the representative SCN LFP raw traces, LFP power, and LFP power spectrogram in a mouse before and after the application of PWL demonstrated that there was no change in the LFP amplitude or frequency and power between pre-PWL and post-PWL. However, a representative LFP recorded during continuous inhalation of 2.5% sevoflurane (Figure 3K) showed that the LFP amplitude decreased and the LFP frequency increased after the application of MBL. A spectrogram computed from the same animal in Figure 3K showed that the LFP power after the application of MBL was higher than pre-MBL (Figures 3L,M). Therefore, we conclude that the application of MBL could activate SCN neurons during sevoflurane anesthesia.

### Lesion of SCN Neurons Abolished Monochromatic Blue Light-Induced Arousal-Promoting Responses During Sevoflurane Anesthesia

**Figure 4:**
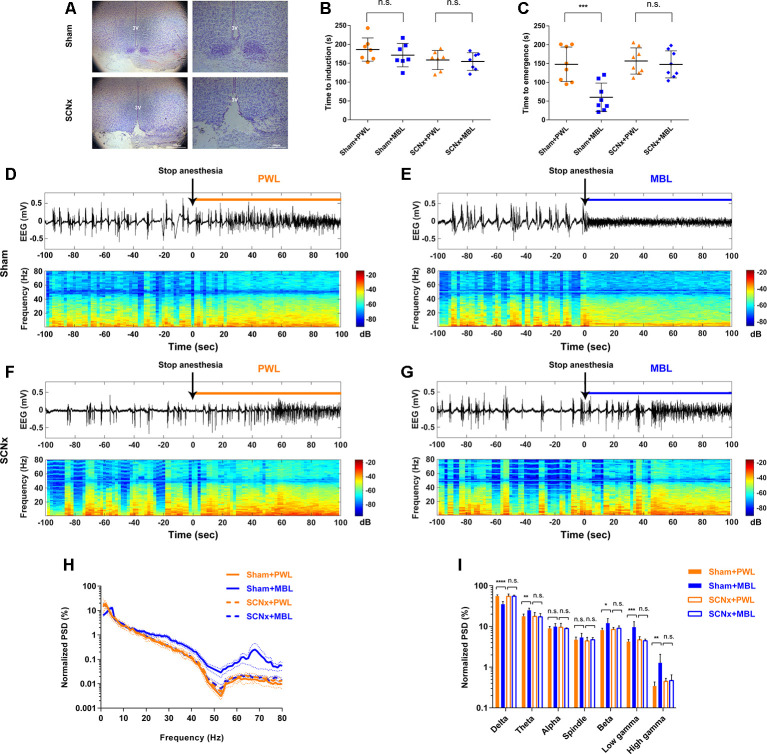
Lesion of SCN neurons abolished MBL-induced arousal-promoting responses during sevoflurane anesthesia. **(A)** Nissl-stained coronal brain sections of the SCN are shown for the sham (upper) and SCN lesioned (lower) animals. 3V, third ventricle. Scale bar = 200 μm. **(B)** Results of the sevoflurane-inducing LORR time in the sham and SCNx groups (SCN lesioned), both of which received PWL or MBL, respectively. The sham animals treated with MBL showed no statistically significant difference in induction time relative to the sham-PWL mice (PWL, 186.10 ± 30.67 s vs. MBL, 171.30 ± 31.01 s) in sevoflurane anesthesia. The SCNx animals treated with MBL did not show a significant difference in induction times relative to those treated with PWL (158.40 ± 25.41 s vs. MBL, 154.60 ± 23.23 s) (n.s. = not significant, *n* = 7 in each group, *F*_(3,24)_ = 1.848, ordinary one-way ANOVA, and Tukey’s multiple comparisons test). **(C)** The sham animals treated with MBL showed a significant decrease in recovery time relative to the sham-PWL mice (PWL, 147.90 ± 46.01 s vs. MBL, 60.13 ± 37.84 s) in sevoflurane anesthesia. The SCNx animals treated with MBL did not show a significant difference in recovery times relative to those treated with PWL (PWL, 156.80 ± 34.85 s vs. MBL, 147.60 ± 35.96 s; ***p < 0.001 compared with sham PWL, n.s. = not significant, *n* = 8 in each group, *F*_(3,28)_ = 10.94, ordinary one-way ANOVA, and Tukey’s multiple comparisons test). Data are expressed as mean ± SD. **(D–G)** Representative electroencephalogram traces (upper) and electroencephalogram power spectrogram (lower) in the sham **(D,E)** and SCNx groups (SCN lesioned; **F,G)**, both of which received PWL or MBL, respectively. Time 0 indicates stopping sevoflurane anesthesia and the beginning of light stimulation. The orange and blue lines indicate the 100 s intervals used for power spectral density comparisons. **(H)** Normalized group PSD from the sham PWL (orange solid line), sham MBL (blue solid line), SCNx PWL (orange dotted line), and SCNx MBL (blue dotted line) groups. **(I)** The sham animals treated with MBL showed a significant decrease in the relative electroencephalogram delta power and an increase in the theta, beta, low gamma, and high gamma power compared to the sham-PWL mice. The SCNx animals treated with MBL showed no significant difference in electroencephalogram power relative to those treated with PWL (*p < 0.05, **p < 0.01, ***p < 0.001, and ****p < 0.0001 compared with sham PWL, n.s. = not significant, *n* = 6 in each group, ordinary one-way ANOVA, and Tukey’s multiple comparisons test). Data are expressed as mean ± SD. PWL, polychromatic white light; MBL, monochromatic blue light.

To study the role of SCN in the arousal-promoting effect of MBL under sevoflurane anesthesia, we performed bilateral ablation of the SCN using a sheathed platinum-iridium lesion electrode with a current pulse of 2.5 mA for 20 s. The cell death of the ablated SCN was confirmed by Nissl staining (Figure 4A). Three mice were removed from the study because they did not show normal visual function after ablation of the SCN. The mice that showed normal vision and proved to have complete lesions were included for the SCN lesioned group. After 1 week of recovery, the time to induction and time to emergence was tested. As shown in Figure 4B (*F*_(3,24)_ = 1.848), SCN ablation did not alter the induction time between the PWL and MBL groups (SCNx PWL, 158.40 ± 25.41 s vs. SCNx MBL, 154.60 ± 23.23 s, *p* > 0.05). Moreover, the difference in the emergence time between PWL and MBL in sham mice disappeared when the SCN neurons were ablated (Figure 4C, *F*_(3,28)_ = 10.94, SCNx PWL, 156.80 ± 34.85 s vs. SCNx MBL, 147.60 ± 35.96 s, *p* > 0.05). These results suggest that the SCN is important for the arousal-promoting effect of MBL under sevoflurane anesthesia.

To further confirm that the SCN plays a key role in the arousal-promoting effect of MBL under sevoflurane anesthesia, we analyzed cortical EEG activity for a duration of 100 s after stopping sevoflurane anesthesia. In the SCN lesioned group, differences in EEG traces, EEG power spectrogram, and normalized group PSD between PWL and MBL in the sham mice (Figures 4D,E,H,I) were not observed (Figures 4F–I). The application of MBL in the SCN-ablated mice did not produce a wake-promoting response in behavioral tests and EEG recordings, indicating that lesions in the SCN reversed the behavioral and cortical arousal from sevoflurane anesthesia induced by MBL.

### Lesion of SCN Neurons Reversed the Decrease in the Burst Suppression Ratio Induced by Monochromatic Blue Light During Continuous Inhalation of 2.5% Sevoflurane

**Figure 5:**
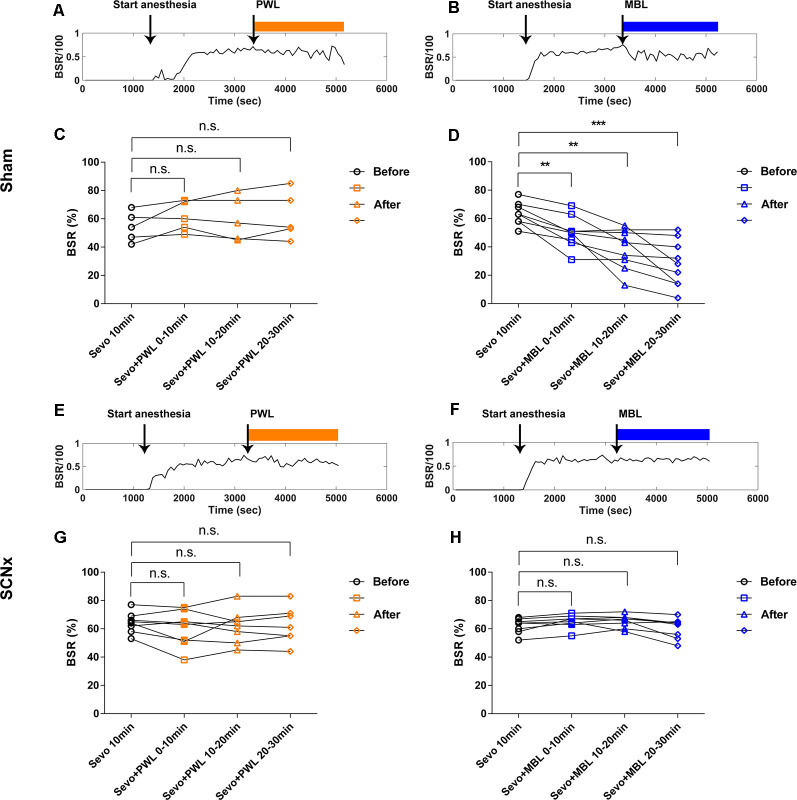
Lesion of SCN neurons reversed the decrease of the burst suppression ratio induced by MBL during continuous inhalation of 2.5% sevoflurane. **(A,B)** Typical examples of the BSR response under sevoflurane anesthesia in the sham group-administered PWL **(A)** or MBL **(B)**. The orange and blue boxes indicate the duration of light exposure. **(C,D)** Effect of PWL **(C)** and MBL **(D)** on the electroencephalogram BSR during continuous inhalation of 2.5% sevoflurane in the sham group. The sham mice treated with MBL showed a significant 21% decrease (**) in the BSR during the first 10 min post-MBL and a 39% decrease (**) during the second 10 min post-MBL and a 56% decrease (***) during the last 10 min post-MBL compared to the sham pre-MBL 10 min (**p < 0.01 and ***p < 0.001 compared with sham pre-MBL 10 min, *n* = 9, RM one-way ANOVA, and Tukey’s multiple comparisons test with the Greenhouse-Geisser correction, *F*_(1.765,14.12)_ = 23.07). However, the sham-PWL-treated animals showed no significant difference in the BSR compared to sham pre-PWL (n.s. = not significant, compared with sham pre-PWL 10 min, *n* = 5, RM one-way ANOVA, and Tukey’s multiple comparisons test with the Greenhouse-Geisser correction, *F*_(1.180,4.719)_ = 1.340). **(E,F)** Typical examples of the BSR response under sevoflurane anesthesia in the SCNx group-administered PWL **(E)** or MBL **(F)**. The orange and blue boxes indicate the duration of light exposure. **(G,H)** Effect of PWL **(G)** and MBL **(H)** on electroencephalogram BSR during continuous inhalation of 2.5% sevoflurane in the SCNx group. The SCNx mice treated with MBL showed no significant difference in BSR compared to the SCNx pre-MBL 10 min (n.s. = not significant compared with SCNx pre-MBL 10 min, *n* = 8, RM one-way ANOVA, and Tukey’s multiple comparisons test with the Greenhouse-Geisser correction, *F*_(1.550,10.85)_ = 3.516). SCNx PWL-treated animals showed no significant difference in the BSR compared to SCNx pre-PWL (n.s. = not significant compared with SCNx pre-PWL 10 min, *n* = 8, RM one-way ANOVA, and Tukey’s multiple comparisons test with the Greenhouse-Geisser correction, *F*_(1.775,12.42)_ = 1.005). PWL, polychromatic white light; MBL, monochromatic blue light.

To further confirm that the SCN plays a crucial role in the arousal-promoting effect of MBL under sevoflurane anesthesia, we observed the effect of lesions in the SCN on the BSR during continuous inhalation of 2.5% sevoflurane with the application of MBL. In the sham group, consistent with the previous results, the application of MBL induced a significant 21% decrease (*p* < 0.01) in the BSR during the first 10 min post-MBL, a 39% decrease (*p* < 0.01) during the second 10 min post-MBL, and a 56% decrease (*p* < 0.001) during the last 10 min post-MBL compared to the pre-MBL 10 min (Figures 5B,D, *F*_(1.765,14.12)_ = 23.07), while the application of PWL showed no significant difference in the BSR compared to pre-PWL (Figures 5A,C, *F*_(1.180,4.719)_ = 1.340, *p* > 0.05). In the SCN lesioned group, the SCN lesions reversed the decrease in the BSR during continuous inhalation of 2.5% sevoflurane induced by MBL. As shown in Figures 5F,H, compared to before the application of MBL, the application of MBL had no significant impact on the BSR in the SCN-ablated mice (*F*_(1.550,10.85)_ = 3.516, *p* > 0.05). Lesions in the SCN neurons did not affect the BSR between the pre-PWL and post-PWL groups (Figures 5E,G, *F*_(1.775,12.42)_ = 1.005, *p* > 0.05).

**Figure 6:**
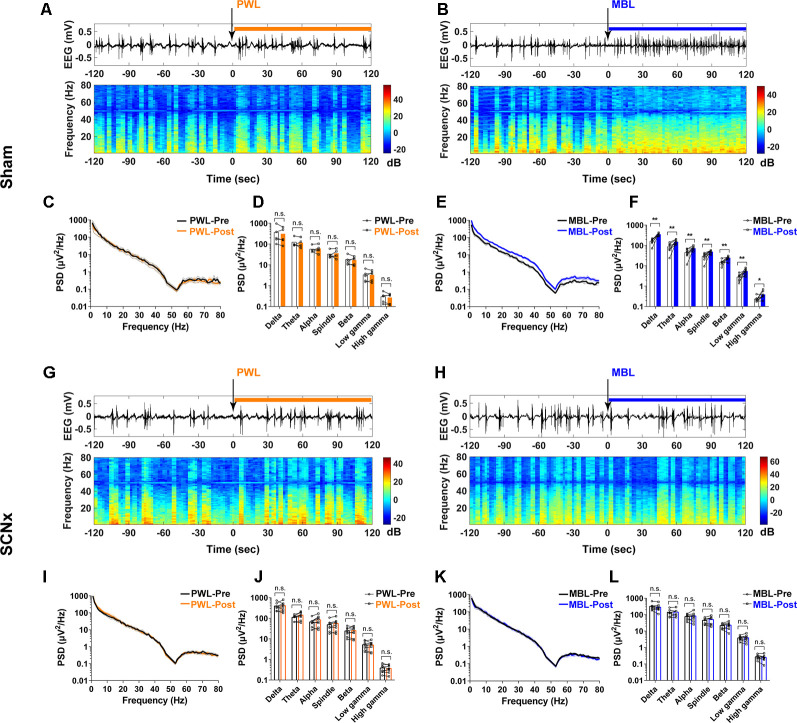
Lesion of SCN neurons eliminated the increase in cortical EEG activity induced by MBL during continuous inhalation of 2.5% sevoflurane. **(A,B)** Representative electroencephalogram traces (upper) and electroencephalogram power spectrogram (lower) in the sham group-administered PWL **(A)** or MBL **(B)**. Time 0 indicates the beginning of light stimulation during continuous sevoflurane anesthesia. The orange and blue lines indicate the 120 s intervals used for power spectral density comparisons. **(C)** PSD in the sham mice computed from 2 min before PWL (black) and 2 min after PWL (orange). **(D)** The sham mice treated with PWL showed no statistically significant difference in electroencephalogram power compared to sham pre-PWL (n.s. = not significant compared with sham pre-PWL, *n* = 5, paired Student’s *t*-tests). **(E)** PSD in the sham mice computed from 2 min before MBL (black) and 2 min after MBL (blue). **(F)** The sham mice treated with MBL showed increased electroencephalogram power in all of the frequency bands compared to sham pre-MBL (*p < 0.05 and **p < 0.01 compared with sham pre-MBL, *n* = 8, and paired Student’s *t*-tests). **(G,H)** Representative electroencephalogram traces (upper) and electroencephalogram power spectrogram (lower) in the SCNx group-administered PWL **(G)** or MBL **(H)**. Time 0 indicates the beginning of light stimulation during continuous sevoflurane anesthesia. The orange and blue lines indicate the 120 s intervals used for power spectral density comparisons. **(I)** PSD in the SCNx mice computed from 2 min before PWL (black) and 2 min after PWL (orange). **(J)** The SCNx mice treated with PWL showed no statistically significant difference in electroencephalogram power compared to SCNx pre-PWL (n.s. = not significant compared with SCNx pre-PWL, *n* = 8, and paired Student’s *t*-tests). **(K)** PSD in the SCNx mice computed from 2 min before MBL (black) and 2 min after MBL (blue). **(L)** The SCNx mice treated with MBL showed no statistically significant difference in electroencephalogram power compared to SCNx pre-MBL (n.s. = not significant, compared with SCNx pre-MBL, *n* = 8, and paired Student’s *t*-tests). PWL, polychromatic white light; MBL, monochromatic blue light.

We examined the cortical EEG activity as previously described to determine the effects of SCN lesions on EEG power and the BSR with the application of MBL. In the sham group, the power at all frequency bands significantly increased after MBL exposure (*p* < 0.01; Figures 6B,E,F), while the mice treated with PWL showed no statistically significant difference in EEG power compared to pre-PWL (*p* > 0.05; Figures 6A,C,D). However, in the SCN lesioned group, MBL did not drive EEG changes in EEG traces, EEG power spectrograms (Figure 6H), and group PSD (*p* > 0.05; Figures 6K,L). Additionally, SCN lesions did not affect the EEG power between the pre-PWL and post-PWL groups (*p* > 0.05; Figures 6G,I,J). These results showed that the SCN lesions reversed the decrease in the BSR induced by MBL during continuous inhalation of 2.5% sevoflurane.

### Monochromatic Blue Light Increased c-Fos Expression in the LH and PFC but Not in the LC and VLPO During Sevoflurane Anesthesia

**Figure 7:**
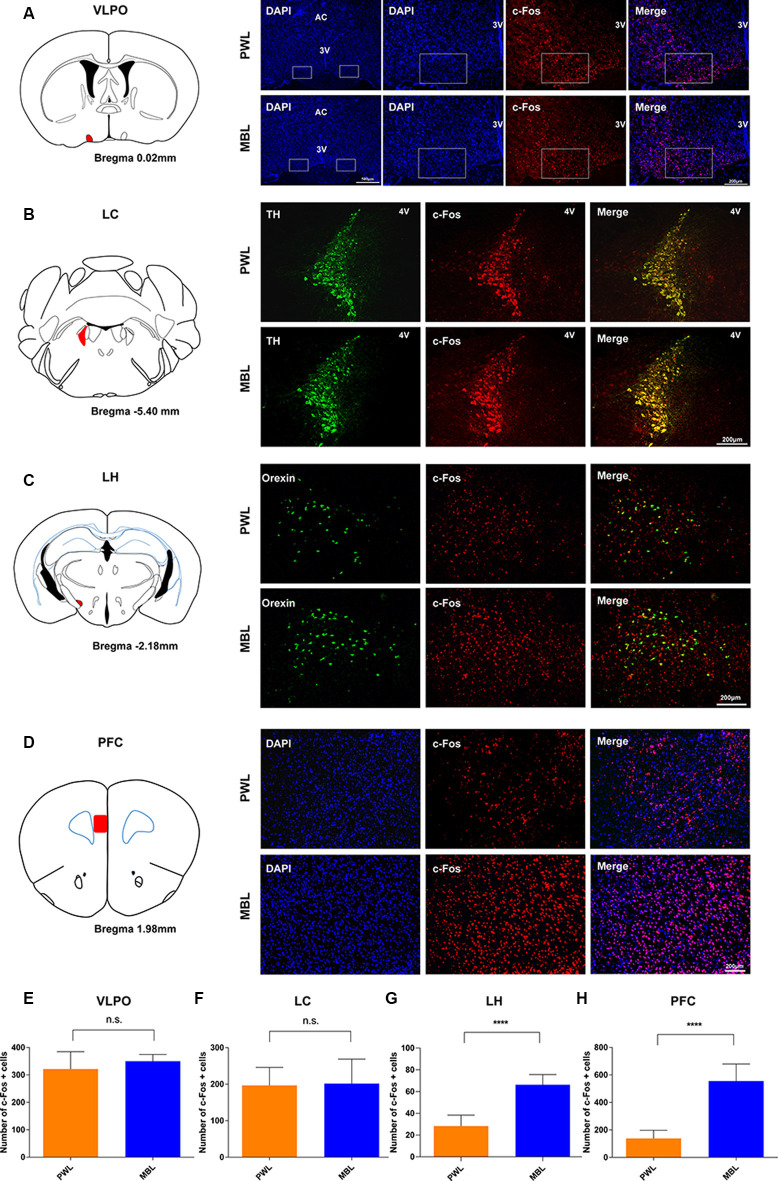
MBL increased c-Fos expression in the LH and PFC but not in the LC and VLPO during sevoflurane anesthesia. **(A–D)** Effect of PWL and MBL on c-Fos expression in the VLPO (right in panel **A**), LC (right in panel **B**), LH (right in panel **C**), and PFC (right in panel **D**) during sevoflurane anesthesia. (Left in panel **A**) Position of the VLPO in the mouse brain atlas. The red indicates the VLPO. (Left in panel **B**) Position of the LC in the mouse brain atlas. The red indicates the LC. (Left in panel **C**) Position of the LH in the mouse brain atlas. The red indicates the LH. (Left in panel **D**) Position of the PFC in the mouse brain atlas. The red indicates the PFC. 3V, third ventricle. 4V, fourth ventricle. AC, anterior commissure. Scale bar = 200 μm. **(E–H)** Quantitative analysis of the number of c-Fos-positive cells in the PWL and MBL groups. The MBL group showed an increase in c-Fos expression compared to the PWL group in areas of the LH and PFC (****p < 0.0001 compared with PWL, *n* = 6–8 in each group, and unpaired Student’s *t*-tests with Welch’s correction). The MBL-treated animals showed no statistically significant difference in VLPO and LC c-Fos expression compared to the PWL-treated animals. Data are expressed as mean ± SD (n.s. = not significant, compared with PWL, *n* = 6–8 in each group, and unpaired Student’s *t*-tests with Welch’s correction). VLPO, ventrolateral preoptic area. LC, locus coeruleus. LH, lateral hypothalamus. PFC, the prefrontal cortex. PWL, polychromatic white light; MBL, monochromatic blue light.

To further explore the mechanism of the arousal-promoting effect of MBL under sevoflurane anesthesia, the numbers of c-Fos-positive neurons in the SCN downstream area were determined after the application of MBL or PWL. As shown in Figures 7A,E, the application of MBL to the mice showed no statistically significant difference in c-Fos expression in sleep-promoting nucleus ventrolateral preoptic areas (VLPO; *p* = 0.2632, *t* = 1.193, and *df* = 9.024). In addition, c-Fos expression in the locus coeruleus (LC) was not significantly different in the MBL and PWL groups (Figures 7B,F, *p* = 0.8858, *t* = 0.1476, and *df* = 9.183). Interestingly, the application of MBL elicited a significant increase in c-Fos expression in the lateral hypothalamus (LH) orexinergic neurons (Figures 7C,G, *p* < 0.0001, *t* = 7.795, and *df* = 14). We found that c-Fos expression in the PFC also increased in the MBL group compared with the PWL group (Figures 7D,H, *p* < 0.0001, *t* = 8.338, and *df* = 10.59), consistent with the previous EEG results that MBL could increase cortical EEG activity under sevoflurane anesthesia. These results suggest that the application of MBL increases the activation of cortical and subcortical SCN downstream arousal nuclei, which may accelerate the transition from general anesthesia to an arousal state.

## Discussion

In this study, we demonstrated that MBL led to a faster emergence from sevoflurane anesthesia and accelerated cortical arousal during the sevoflurane emergence period. MBL decreased the BSR and increased cortical EEG activity. Moreover, MBL increased the activity of the SCN neurons under sevoflurane anesthesia. Lesion of SCN reversed MBL-induced arousal from sevoflurane anesthesia and abolished MBL-induced decreases in the BSR. MBL activated the SCN projecting region including LH and PFC, but not LC, and MBL did not affect sleep-promoting VLPO under sevoflurane anesthesia. Taken together, these results provide evidence that MBL promotes arousal from sevoflurane anesthesia *via* activating the SCN region and its multiple downstream awake-promoting nuclei.

Emerging evidence has demonstrated that there are two systems for detecting light in mammals and humans, image- and NIF visual systems (Legates et al., 2014). The physiological, behavioral, and cognitive functions that are modulated by light but not associated with conscious image perception are called NIF functions (Daneault et al., 2016). The NIF functions are mediated by melanopsin, which is expressed by ipRGCs, and then are projected to the SCN of the anterior hypothalamus, the master circadian endogenous pacemaker (Berson et al., 2002; Gooley et al., 2003). The SCN sends signals to the hypothalamic and non-hypothalamic nuclei regulating circadian rhythms, sleep and wake cycles, hormone release, learning, and memory (Lucas et al., 1999; Altimus et al., 2008; Vandewalle et al., 2009; Golombek and Rosenstein, 2010; Gooley et al., 2012). It is expected that brain activities are silenced during general anesthesia, and the brain’s responsiveness to external stimuli is lost as anesthesia deepens (Ries and Puil, 1993; Detsch et al., 1999). However, animal and human studies have demonstrated that brain activities responsive to somatosensory (Yli-Hankala et al., 1993), auditory (Land et al., 2012), and visual stimuli (Hartikainen et al., 1995; Hudetz and Imas, 2007; Aggarwal et al., 2019) remain preserved under general anesthesia. Consistent with previous studies, the EEG and LFP results demonstrated that MBL decreased BSR and increased cortical and SCN activity during continuous inhalation of 2.5% sevoflurane. This is further evidence that non-visual neural light pathways are preserved under sevoflurane anesthesia.

MBL at wavelengths between 400 nm and 490 nm has both beneficial and harmful effects on living organisms. High-energy short-wave blue light between 415 and 455 nm is the most harmful, causing photochemical retinal damage. However, wavelengths between 460 and 480 nm are absorbed by ipRGCs engaged in the regulation of NIF functions (Tosini et al., 2016; Zhao et al., 2018). Moderate blue light exposure is beneficial for living organisms. It can treat the seasonal affective disorder (SAD; Glickman et al., 2006; Meesters et al., 2011), improve alertness, performance, and cognition (Viola et al., 2008; Motamedzadeh et al., 2017; Scheuermaier et al., 2018) attenuate inflammation and organ injury (Yuan et al., 2016), and enhance bacterial clearance and its intrinsic antimicrobial properties (Wang et al., 2017; Lewis et al., 2018). MBL also can regulate sleep to some extent *via* melanopsin ipRGC non-visual neural pathways. In animal studies, blue light increases behavioral arousal *via* M1 ipRGCs projecting to the SCN in the hypothalamus (Bourgin and Hubbard, 2016; Pilorz et al., 2016). In human studies, evening exposure to MBL reduces SWA and shortens REM sleep (Munch et al., 2006). Consistent with these studies, our behavioral study found that MBL shortened the emergence time but had no effect on the induction time, suggesting that MBL led to faster emergence from sevoflurane anesthesia. The EEG results showed that MBL decreased the relative EEG delta power and increased the spindle, beta, low gamma, and high gamma power, demonstrating that MBL accelerated cortical arousal during sevoflurane emergence. This further confirmed that MBL can produce arousal-promoting effects during anesthesia.

The SCN regulates the sleep-wake cycle through arousal mechanisms that oppose the homeostatic drive for sleep in mammals (Saper et al., 2001; Easton et al., 2004). Lesions in the SCN can disrupt the circadian sleep-wake cycle and significantly reduce daily time awake in squirrel monkeys (Edgar et al., 1993). Recent research also demonstrated that the SCN plays a critical role in arousal-promoting response to blue light. Mice exposed to blue light showed greater c-Fos expression in the SCN (Pilorz et al., 2016). Consistent with the data presented herein, our study found that MBL exposure under sevoflurane anesthesia elicited a significant increase in c-Fos expression in the SCN and considerably increased SCN LFP activity. We found that the SCN lesions significantly abolished the arousal-promoting effects of MBL and reversed MBL-induced decreases in the BSR, implying that SCN plays a key role in regulating the arousal-promoting effects of MBL under sevoflurane anesthesia. Several brain neurotransmitter or neuromodulator systems, including cholinergic, orexinergic, and noradrenergic, have been strongly involved in regulating arousal (Brown et al., 2012; Saper and Fuller, 2017). It is well established that light stimulates melanopsin ipRGCs, directly conveying signals to the VLPO mediating sleep responses or transmitting signals to the SCN, and then the SCN sends efferent projections to the hypothalamic and non-hypothalamic structures, including the dorsomedial nucleus of the hypothalamus (DMH), the paraventricular nucleus of the hypothalamus (PVN), LH, LC, and BF, regulating wakefulness (Aston-Jones et al., 2001; Legates et al., 2014; Daneault et al., 2016; Szabadi, 2018). In the SCN downstream area, we found that MBL increased c-Fos expression in the LH and PFC but not in the LC. Recent studies indicated that orexinergic neurons can be directly activated by light through ipRGC projections (Hattar et al., 2006) or maybe indirectly activated by light *via* the SCN (Abrahamson et al., 2001). A light pulse also increased LH c-Fos expression in the diurnal rodent Nile grass rat (Adidharma et al., 2012). Previously published studies demonstrated that LH and PFC play essential roles in arousal from general anesthesia (Kelz et al., 2008; Pal et al., 2018). Thus, our results are further supported. Prior research reported that sevoflurane could directly excite rat LC neurons (Yasui et al., 2007). Because the LC neurons have already been activated by exposure to sevoflurane, MBL would not be able to further increase the activity of LC cells. This may explain why MBL did not affect LC c-Fos expression under sevoflurane anesthesia. Additionally, consistent with our results, a previous study showed that systemic noradrenergic reuptake blockers could not promote behavioral arousal during propofol anesthesia (Kenny et al., 2015). Since ipRGCs directly convey the signal to the VLPO and the SCN also indirectly sends projections to the VLPO (Chou et al., 2003; Gooley et al., 2003), we observed the effect of MBL on VLPO c-Fos expression under sevoflurane anesthesia. Interestingly, we found that MBL did not affect VLPO c-Fos expression under sevoflurane anesthesia. This likely occurred due to the activation of VLPO neurons during general anesthesia (Moore et al., 2012; Mccarren et al., 2014), and this activation masked the biological effects of MBL. These results are in agreement with Milosavljevic et al. (2016). They found that chemogenetic activation of melanopsin retinal ganglion cells resulted in increased c-Fos expression in multiple nuclei such as DMH, LH, and PVT but had no effect on the expression of VLPO c-Fos (Milosavljevic et al., 2016).

## Clinical Significance

There are currently no clinically approved interventions that can be used routinely to promote emergence from general anesthesia. As a non-pharmacologic approach, MBL can lead to faster emergence from sevoflurane anesthesia by decreasing the depth of anesthesia. In laboratory animal research and pilot human feasibility trials, bright blue light influenced immunity, enhanced bacterial clearance, attenuated inflammation, and reduced oxidative stress (Choi et al., 2012; Yuan et al., 2016; Lewis et al., 2018). Ibrahim et al. (2017) also reported that blue light can result in thermal analgesia in naive rats. Zhou et al. (2018) found that pharmacogenetic activation of mouse orexin neurons facilitated emergence from isoflurane anesthesia and increased pain tolerance. In our study, we found that MBL excited LH neurons under sevoflurane anesthesia, thus we infer that MBL exposure during sevoflurane anesthesia might also have had an antinociceptive effect *via* activation of LH orexin neurons. Using environmental blue light during anesthesia recovery may promote the emergence and strengthen analgesia, which is worth further study.

## Limitations

In this study, we did not investigate the effects of MBL exposure on sevoflurane MAC for induction (MAC_LORR_) and emergence (MAC_RORR_). In immunofluorescent staining, we found that the application of MBL under sevoflurane anesthesia increased c-Fos expression in the PFC, LH, and SCN, while a further specific neuron confirmation is needed. SCN LFP recordings and bilateral SCN electrical lesions are rough techniques, and elaborate and selective methods to further analyze the function of SCN-specific neurons are needed. Additionally, SCN electrical lesions might destroy neuronal projections from the retina to other brain areas. Thus, whether other neuronal projections from the retina are intact in SCN lesion mice need to be observed. We did not examine the effect of SCN lesions on the MBL-induced changes in c-Fos in the LH and PFC, so further studies are needed. In the present study, we focused only on male mice, which may make our results unrepresentative of the species in general. Light has obvious biological differences between nocturnal and diurnal subjects, so the relevance of our results remains to be determined in clinical settings.

## Conclusion

In summary, these findings support the hypothesis that MBL plays a role in promoting arousal in sevoflurane anesthesia.

## Data Availability Statement

The raw data supporting the conclusions of this article will be made available by the authors, without undue reservation.

## Ethics Statement

The animal study was reviewed and approved by Experimental animal ethics committee of Tongji Hospital Affiliated to Tongji Medical College of Huazhong University of science and technology.

## Author Contributions

WM and BT: study conception, behavioral tests and data interpretation. SZ YH, and JW: immunofluorescence experiments. DL and JL: EEG recording and analysis. DL, JD, and XC: data analysis. DL and WM: drafting of the manuscript. DL, WM, and BT: critical revision of the manuscript. WM: obtained funding.

## Conflict of Interest

The authors declare that the research was conducted in the absence of any commercial or financial relationships that could be construed as a potential conflict of interest.

## Footnotes

^1^http://chronux.org/

## Supplementary Material

FIGURE S1 The spectrum distribution of polychromatic white light (PWL).

FIGURE S2 Schematic of the experimental setup. Schematic shows a modified experimental setup in which the animal can freely behave while EEG data is recorded and the light condition is performed during sevoflurane general anesthesia.

## Abbreviations

BSR: burst-suppression ratio
ipRGCs: intrinsically photosensitive retinal ganglion cells
LFP: local field potential
LORR: loss of righting reflex
MAC: minimum alveolar concentration
MBL: monochromatic blue light
PWL: polychromatic white light
RORR: recovery of righting reflex
SCN: suprachiasmatic nucleus.
