# Supplementary figures and images for: Monochromatic Blue Light Activates Suprachiasmatic Nucleus Neuronal Activity and Promotes Arousal in Mice Under Sevoflurane Anesthesia
Source: Front Neural Circuits. 2020 Aug 18;14:55. doi: 10.3389/fncir.2020.00055 (PMC7461971; doi:10.3389/fncir.2020.00055)

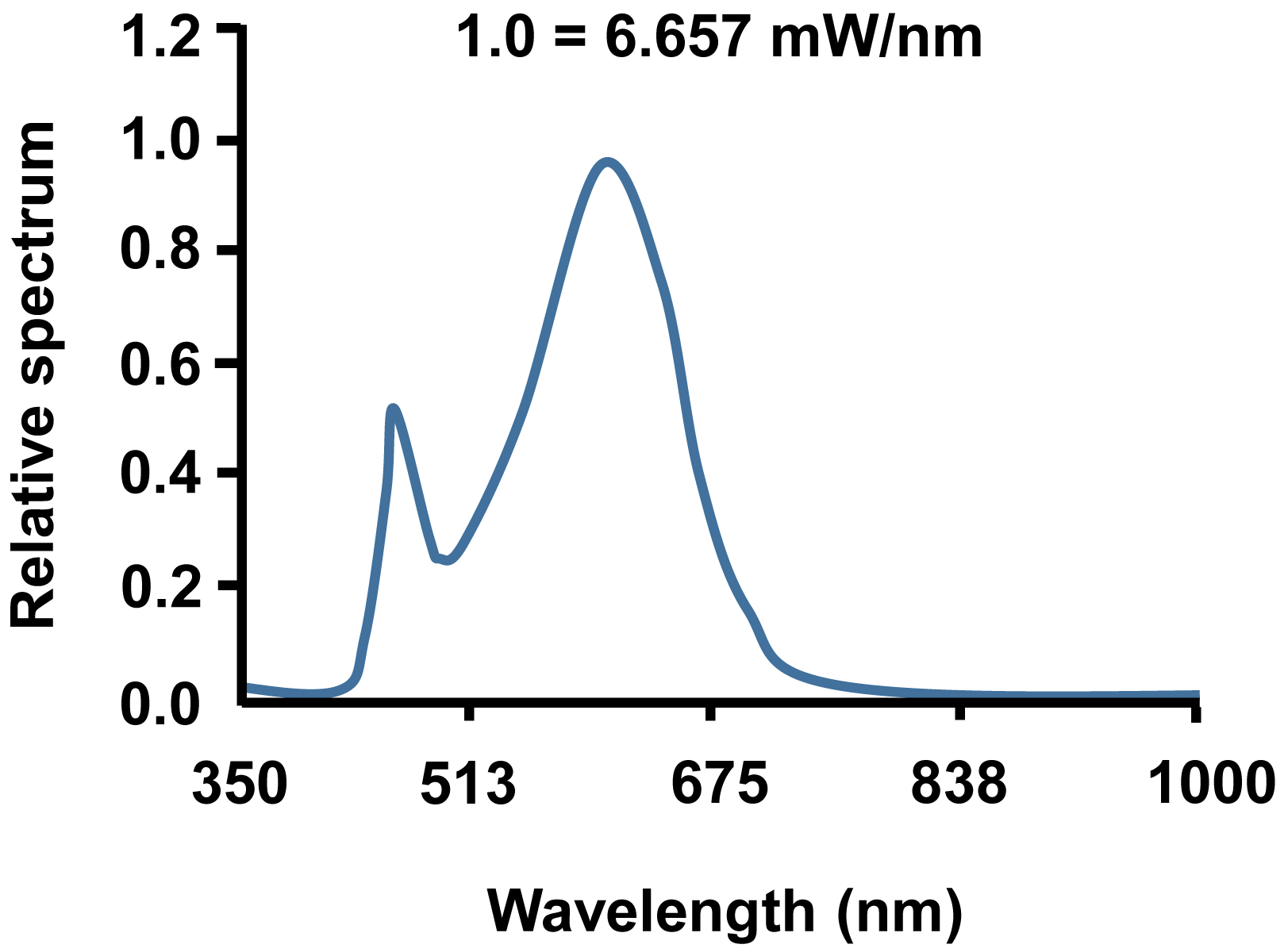

Supplement: FIGURE S1 — The spectrum distribution of polychromatic white light (PWL). [file Image_1.TIF]

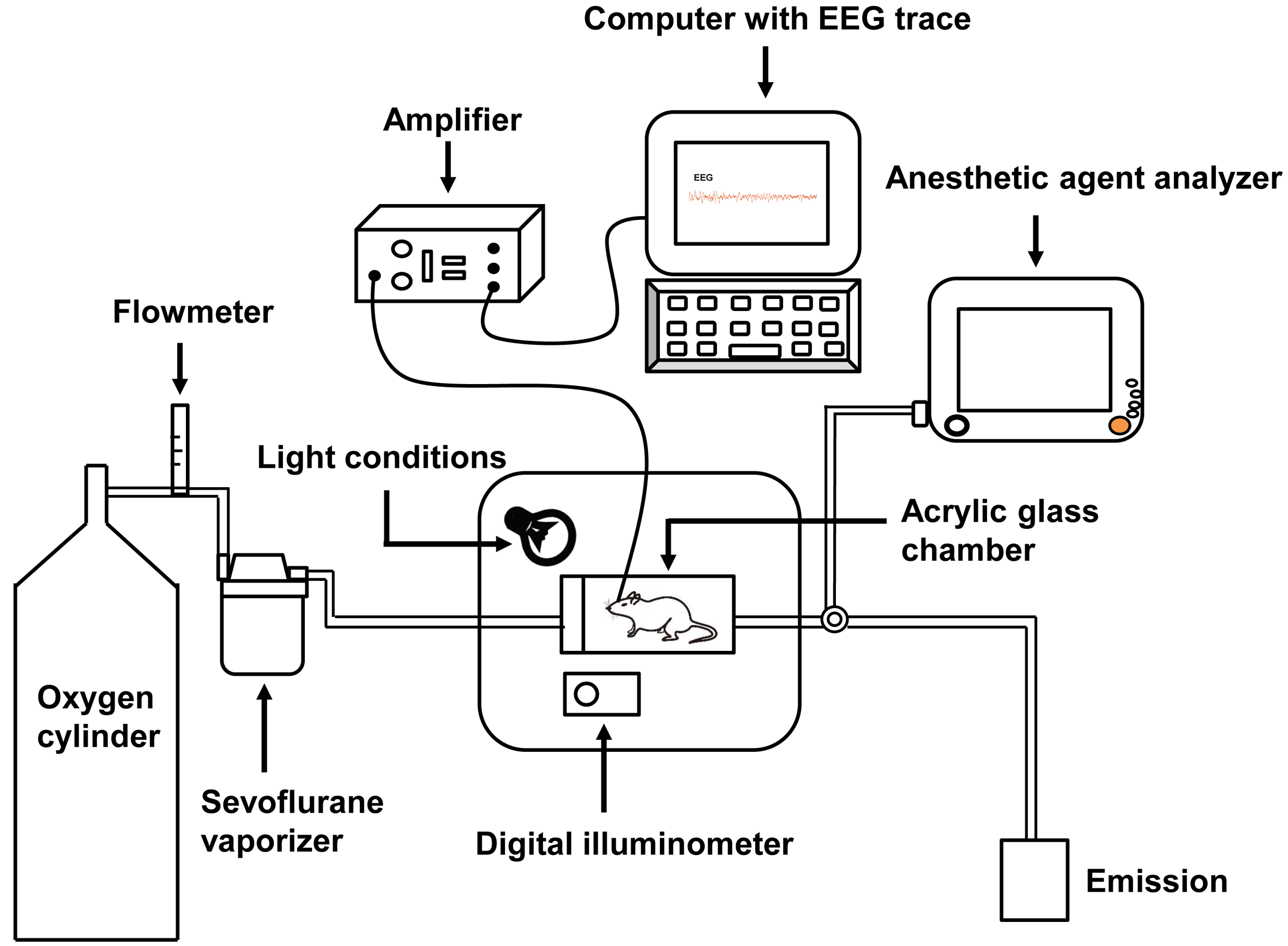

Supplement: FIGURE S2 — Schematic of the experimental setup. Schematic shows a modified experimental setup in which the animal can freely behave while EEG data is recorded and the light condition is performed during sevoflurane general anesthesia. [file Image_2.TIF]
